# The effect of standing and sitting postures on breathing in brass players

**DOI:** 10.1186/2193-1801-3-210

**Published:** 2014-04-28

**Authors:** Kevin Price, Philippe Schartz, Alan HD Watson

**Affiliations:** Royal Welsh College of Music and Drama, Cardiff, UK; BBC National Orchestra of Wales, Hoddinott Hall, Cardiff, UK; School of Biosciences, Cardiff University, Museum Avenue, Cardiff, CF10 3AX Wales UK

**Keywords:** Respiratory movements, Abdominal muscles, Musician, Brass, Seating

## Abstract

**Purpose:**

The object of this study was to examine the effect of posture on breathing in brass players. Breathing when standing was compared with sitting erect on a flat, downward or upward sloping seat, or on a reclining seat.

**Methods:**

Spirometry was used to measure aspects of lung function. Muscle activity and respiratory movements during different playing tasks were recorded using electromyography and inductive plethysmography.

**Results:**

Only sitting in a reclining position produced statistically significantly lower values for VC, FVC, FEV1, PEF than standing. When players were asked to produce a note of maximum duration, only a downward sloping seat caused a significant change (an 11% reduction) compared to standing. When seated, the abdominal component of respiratory movement was significantly higher during these long notes than when standing, though maximum activity in abdominal wall muscles was significantly reduced (by 32–44%). On a downward sloping seat, muscle activity was significantly higher (9%) than on a flat seat. Tongued and untongued sforzando notes recruited significantly less abdominal muscle activity (33–67%) when sitting than when standing. When playing a trumpet study, abdominal muscle activity was significantly reduced on a downward sloping seat (by 32%) and on a flat seat (by 40%) in comparison to standing. Muscle activity in the two sitting positions were not significantly different.

**Conclusion:**

Though brass players are often told to “sit as if standing”, abdominal muscle activity is always significantly reduced when sitting on a flat or downward sloping seat, however when greater respiratory effort is required, activity on downward sloping seats may rise closer to that of standing.

## Background

Many pedagogical accounts of breathing in brass players discuss the effect of different standing and sitting postures and though numerous opinions have been expressed about this (Frederiksen 1996; Gordon 1987; Snell 1988; Steenstrup 2004), none has so far been based on experimental evidence. It is often suggested that when standing, vital capacity is greater and breathing movements more free than when sitting i.e. that during inspiration particularly, they require less effort (Steenstrup 2004). If the player does use a chair, they are often instructed to sit “as if you are standing” (Frederiksen 1996) though they may be warned not to sit too erect (Farkas 1956). The type of chair used also has an effect on posture which can in turn have an impact on breathing. It is now quite common for orchestral musicians (including wind players), to play on seats that slope down towards the front in order to improve comfort, particularly when playing for long periods (Horvath 2010; Paull and Harrison 1997). While some modern orchestral chairs have been ergonomically designed to incorporate a degree of slope, the chairs that players are most often presented with do not, and they may therefore adapt them by using wedge shaped cushions or by putting blocks under the back legs. Sitting on a flat seat, reduces or even reverses lumbar lordosis and the rationale for using a sloping seat is the belief that this will restore lumbar curvature to a conformation that more closely resembles standing thus reducing intervertebral disk pressure (see Watson 2009). Whether this can be achieved with the degree of slope that can be readily attained on the platform is rarely discussed; furthermore there has been little consideration of what effect sloping seats might have on breathing. This is the object of the present investigation.

Most studies of the effect of posture on breathing are directed at clinical scenarios, and compare standing with lying supine or more rarely, prone (de Troyer 1983; Kera and Maruyama 2005). This applies even to postural studies of speech and singing (Hoit 1995; Sundberg et al. 1991). Our intention was to examine postures that are more typical of brass musicians and so we compared standing with a range of sitting postures. We first examined the effect of posture on a number of spirometric parameters. We then recorded thoracic and abdominal movements and abdominal muscle activity in brass musicians during a range of playing tasks carried out in different positions. We discovered that posture does have a small but statistically significant influence on spirometric parameters, whose values were consistently greater when standing than in any sitting position. However use of a sloping seat did not show any advantage over a flat one. When playing long notes, abdominal movements and abdominal muscle activity come to dominate as lung volume declines. The degree of inward abdominal movement was greater when sitting than when standing but the level of abdominal muscle activity was actually less. When sitting to play tongued or untongued sforzando blasts or a study piece, abdominal muscle activity was generally less than when standing but there was no significant different between a flat or sloping seat.

## Results

### Experiment 1-spirometry

**Figure 1:**
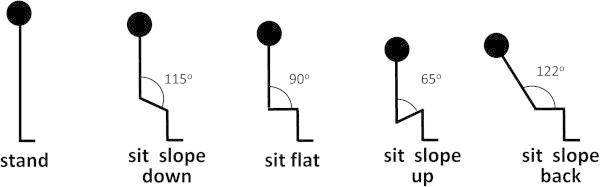
**Sitting positions used in the study.**

The results of the spirometric tests are shown in Table 1. Sitting on a seat with a sloping back (Figure 1) showed the most consistent reduction in respiratory parameters when compared to standing. VC, FVC, FEV1 and PEF were all reduced significantly (p < 0.05). FVC and FEV1 were also significantly reduced compared to all other sitting positions while VC was reduced in comparison to sitting on a flat seat only. The only other significant reduction relative to standing was in VC and FVC in an upward sloping seat, though differences in VC on a downward sloping seat and FVC on a flat seat were close to significance (p = 0.054 and p = 0.052 respectively). For brass players, the most important respiratory parameter is likely to be FVC though the measured task differs from playing an instrument as there is no resistance to airflow and so the PEF values in the test will be much higher than during performance.

**Table 1:** **The effect of standing and different sitting postures on four standard spirometric parameters**

**Vital capacity**				
**Stand**	**Sit flat**	**Sit slope down**	**Sit slope up**	**Slope back**
94.3 ± 2.08%	93.6 ± 1.98%	93.0 ± 2.01%	92.9 ± 1.98%	91.9 ± 1.92%
***Probability***	**Slope back**	**Sit slope up**	**Sit slope down**	**Sit flat**
**Stand**	**0.004**	**0.022**	0.054	0.191
**Sit flat**	**0.050**	0.180	0.201	
**Sit slope down**	0.170	0.466		
**Sit slope up**	0.141			
**Forced Vital Capacity (FVC)**				
**Stand**	**Sit flat**	**Sit slope down**	**Sit slope up**	**Slope back**
99.2 ± 2.2%	98.4 ± 2.00%	98.3 ± 2.03%	98.3 ± 2.18%	96.1 ± 2.18%
***Probability***	**Slope back**	**Sit slope up**	**Sit slope down**	**Sit flat**
**Stand**	**<0.001**	**0.036**	0.167	0.052
**Sit flat**	**0.01**	0.396	0.427	
**Sit slope down**	**0.014**	0.483		
**Sit slope up**	**0.002**			
**FEV1**				
**Stand**	**Sit flat**	**Sit slope down**	**Sit slope up**	**Slope back**
98.3 ± 2.18%	99.2 ± 2.31%	99.1 ± 2.1%	98.5 ± 2.18%	95.7 ± 2.21%
***Probability***	**Slope back**	**Sit slope up**	**Sit slope down**	**Sit flat**
**Stand**	**0.024**	0.355	0.199	0.236
**Sit flat**	**0.001**	0.189	0.262	
**Sit slope down**	**<0.001**	0.158		
**Sit slope up**	**0.001**			
**Peak Expiratory Flow (PEF)**				
**Stand**	**Sit flat**	**Sit slope down**	**Sit slope up**	**Slope back**
88.0 ± 1.94%	85.6 ± 1.76%	86.3 ± 1.66%	86.1 ± 2.00%	84.8 ± 2.04%
***Probability***	**Slope back**	**Sit slope up**	**Sit slope down**	**Sit flat**
**Stand**	**0.002**	0.080	0.083	0.117
**Sit flat**	0.280	0.391	0.293	
**Sit slope down**	0.105	0.459		
**Sit slope up**	0.116			

For each parameter, the upper cells contain descriptive statistics (mean ± s.e.m., n = 30) expressed as a percentage of predicted values (ECSC), while the lower cells provide a statistical comparison between them. Probabilities of <0.05 are in bold type.

### Experiment 2-standardised playing tasks

This study involved 20 music students who carried out a series of simple standardised playing tasks (long notes and short sforzando notes) while standing and in a variety of sitting positions.

### Note duration

The effects of posture on note duration are shown on Table 2. The length of the notes played when standing at the beginning and end of the series are not significantly different, indicating that the requirement to generate such long notes repeatedly (twelve times during the experiment) did not affect their mean duration. Though the values for the sitting positions were all lower than for standing, this was only statistically significant for sitting on a downward sloping seat (p = 0.025) which resulted in a mean note length that was 11% shorter. When compared to standing, the reduction in mean note length when sitting upright on a flat seat was just outside significance, as was the difference between sitting on a sloping seat and one with a sloping back.

**Table 2:** **Long note duration (mean ± s.e.m. n = 20)**

Stand start	Sit flat	Sit slope down	Slope back	Stand end
27.7 ± 2.4 s	25.9 ± 2.4 s	24.7 ± 2.5 s	25.7 ± 2.5 s	26.4 ± 2.6 s
***Probability***	**Stand end**	**Sit flat**	**Sit slope down**	**Slope back**
**Stand start**	0.183	0.06	**0.025**	0.081
**Sit flat**	0.277	-	0.11	0.411
**Sit slope down**	**0.021**	0.11	-	0.063
**Slope back**	0.157			

Probabilities of <0.05 are in bold type.

### Thoracic and abdominal movement-plethysmography

**Figure 2:**
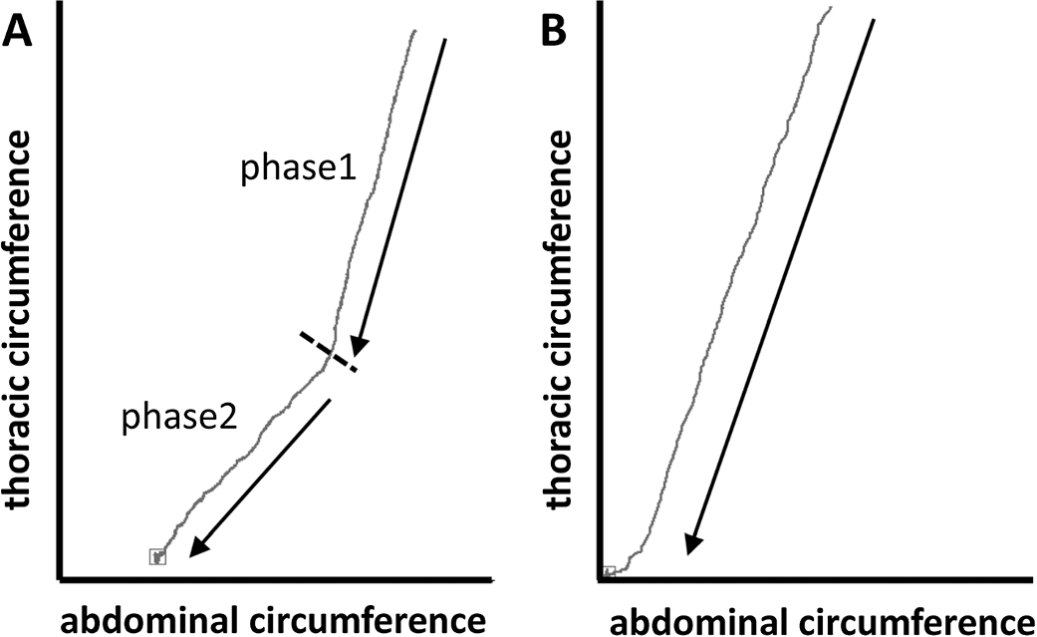
**Konno-Mead traces for the expirations supporting long notes played by two horn players.** The trace from player **A** has two distinct phases with an abrupt transition between them. The second phase has a greater abdominal component. Player **B** shows only one phase, with a constant ratio of thoracic and abdominal involvement throughout the note.

Examples of Konno-Mead plots for the exhalations during notes of maximum duration are shown in Figure 2. In a few individuals these were almost linear, but most showed two distinct phases (Figure 2A,B). The first was steepest, with the reduction in thoracic circumference dominating (indicated by the large positive angles in Table 3). During the second phase the slope of the trace was reduced and there was a greater contribution from the reduction in abdominal circumference (indicated by the lower and sometimes negative angles in Table 4). The two phases were therefore analysed separately. The Konno-Mead plots have no time dimension, but the raw plethysmograph traces from which they were extracted reveal that the time of onset of this second phase varies widely between players, starting between 16–75% of the way through the held note. Posture had no significant impact on the first phase (Table 3), but during the second phase, all sitting positions had a significantly greater abdominal contribution than standing (Table 4). There was however no difference between the sitting postures.

**Table 3:** **Plethysmography long note – phase 1 slope (mean ± s.e.m. n = 20)**

Stand start	Sit flat	Sit slope down	Slope back	Stand end
20.5 ± 3.3°	19.2 ± 3.5°	18.8 ± 3.1°	19.1 ± 2.9°	20.1 ± 3.5°
***Probability***	**Stand end**	**Sit flat**	**Sit slope down**	**Slope back**
**Stand start**	0.441	0.287	0.282	0.318
**Sit flat**	0.255	-	0.413	0.434
**Sit slope down**	0.262	0.413	-	0.457
**Slope back**	0.291			

Probabilities of <0.05 are in bold type.

**Table 4:** **Plethysmography long note – phase 2 slope (mean ± s.e.m. n = 20)**

Stand start	Sit flat	Sit slope down	Slope back	Stand end
9.1 ± 5.9°	-3.7 ± 5.9°	-0.7 ± 4.5°	-3.8 ± 4.8°	9.7 ± 4.1°
***Probability***	**Stand end**	**Sit flat**	**Sit slope down**	**Slope back**
**Stand start**	0.441	**0.022**	**0.016**	**0.007**
**Sit flat**	**0.005**	-	0.220	0.442
**Sit slope down**	**0.004**	0.220	-	0.157
**Slope back**	**0.002**			

Probabilities of <0.05 are in bold type.

### Muscle activity

#### Long notes

**Figure 3:**
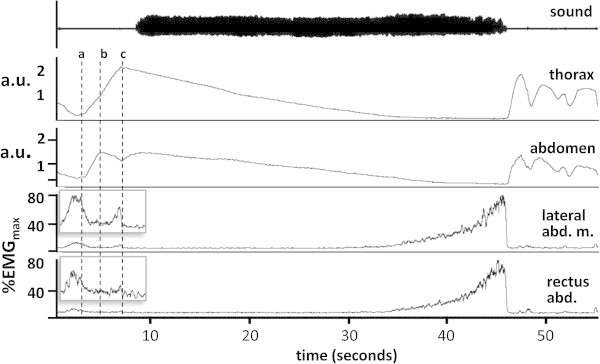
**Abdominal muscle activity (root mean square trace) and movement of the thorax and abdominal wall during a note of maximum duration from a trumpeter.** After inhalation and before the sound is generated a small burst of activity is seen in the external oblique muscle (shown magnified ×10 in the inset). This corresponds to a clear inward movement of the abdomen that will contribute to raising airway pressure to the level needed to initiate the note. During the note, little activity is detectable in the abdominal muscles until near its end.

Before the beginning of the note, two small bursts of activity are often seen in the abdominal muscles (Figure 3). In this case, the small amount of activity in the muscle associated with the end of previous non-playing breath ceases as the thorax and abdomen expand in preparation for playing the long note. The abdomen reaches its maximum expansion first and the external oblique then becomes active again reaching around 5% of maximum voluntary activity (MVC). This produces a noticeable reduction in abdominal circumference even as the thorax continues to expand. Finally, as the thoracic circumference peaks and starts to fall, activity in the external oblique abruptly becomes undetectable and after a short delay, the note is initiated. Several other players showed the same pattern though in some, rectus abdominis was activated in parallel with the external oblique. In others the reduction of chest and abdominal circumference occurred in parallel.

From the beginning of the sustained note, there is a gradual reduction in abdominal circumference indicating that the external oblique and rectus abdominis must be active, however EMG activity is not detectable above background until the final fifty to thirty percent of the exhalation, from which time it rises at an ever increasing rate to reach a peak that is usually over 70% of MVC.

Though for this experiment, EMG activity in the external oblique muscle differed for the standing test at the beginning and end of the series (probably due to fatigue or oxygen debt), both values were significantly greater than the levels recorded in any of the sitting positions (Table 5). The muscle activity on a sloping seat was also significantly greater than for a flat seat. Activity in rectus abdominis in all sitting postures was only about 60% of that when standing (Table 6) which was highly statistically significant, however for this muscle there were no statistical differences between the three sitting positions though the difference between sitting on a sloping and a flat seat was close to significance.

**Table 5:** **Long notes – external oblique activity (% maximum activity, mean ± s.e.m. n = 20)**

Stand start	Sit flat	Sit slope down	Slope back	Stand end
72.1 ± 5.5%	43.4 ± 3.9%	48.8 ± 4.1%	43.7 ± 4.2%	63.5 ± 5.4
***Probability***	**Stand end**	**Sit flat**	**Sit slope down**	**Slope back**
**Stand start**	**0.028**	**<0.001**	**<0.001**	**<0.001**
**Sit flat**	**0.001**	-	**0.033**	0.464
**Sit slope down**	**0.003**	**0.033**	-	0.117
**Slope back**	**0.005**			

Probabilities of <0.05 are in bold type.

**Table 6:** **Long notes – rectus abdominis activity (% maximum activity, mean ± s.e.m. n = 20)**

Stand start	Sit flat	Sit slope down	Slope back	Stand end
75.4 ± 6.3%	42.0 ± 4.3%	43.4 ± 4.8%	44.2 ± 5.0%	71.7 ± 6.0%
***Probability***	**Stand end**	**Sit flat**	**Sit slope down**	**Slope back**
**Stand start**	0.189	**<0.001**	**<0.001**	**<0.001**
**Sit flat**	**<0.001**	-	0.061	0.221
**Sit slope down**	**<0.001**	0.061	-	0.347
**Slope back**	**<0.001**			

Probabilities of <0.05 are in bold type.

#### Short sforzando notes

**Figure 4:**
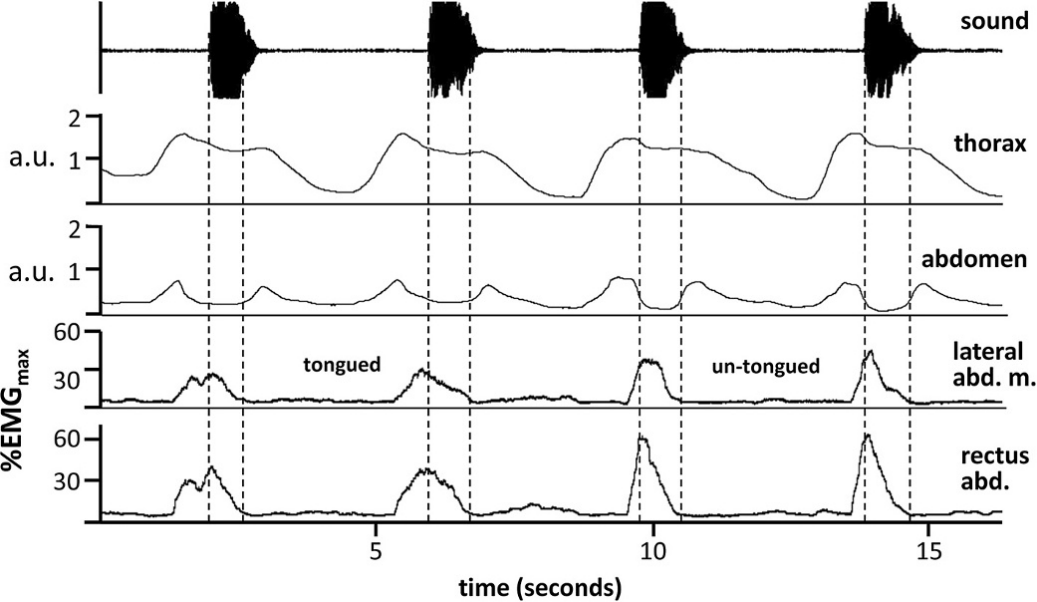
**Abdominal muscle activity and movement of the thorax and abdominal wall during tongued an untongued sforzando notes.** For the tongued notes, the initial increase of muscle activity and the reduction in chest and abdominal circumference occur well before the beginning of the note. For untongued notes these actions are more rapid and of greater magnitude and must be precisely synchronised with note onset. a.u. arbitrary units.

Short sforzando notes are associated with sharply rising bursts of activity in the external oblique and rectus abdominis muscles. For tongued notes, some EMG activity and inward movement of the abdomen occurs before the onset of the sound (Figure 4) because the airflow is initiated by the tongue. In the absence of tonguing, note onset is driven directly by a sharp rise in muscle activity and inward abdominal movement. Whether the notes were tongued or not, both muscles were significantly more active during standing than in all sitting positions (Tables 7, 8, 9 and 10). There was no difference in muscle activity between sitting positions except for the external oblique during untongued notes. In this situation, activity when sitting on a chair with a sloping back was significantly greater than on one with a sloping seat, and close to significance compared to one with a flat seat.

**Table 7:** **Short notes tongued – external oblique (% maximum activity, mean ± s.e.m. n = 20)**

Stand start	Sit flat	Sit slope down	Slope back	Stand end
16.3 ± 3.3%	8.8 ± 2.2%	10.8 ± 4.0%	8.7 ± 2.2%	14.8 ± 3.1%
***Probability***	**Stand end**	**Sit flat**	**Sit slope down**	**Slope back**
**Stand start**	0.176	**0.001**	**0.004**	**0.001**
**Sit flat**	**0.001**	-	0.594	0.265
**Sit slope down**	**0.006**	0.594	-	0.257
**Slope back**	**0.001**			

Probabilities of <0.05 are in bold type.

**Table 8:** **Short notes tongued – rectus abdominis (% maximum activity, mean ± s.e.m. n = 20)**

Stand start	Sit flat	Sit slope down	Slope back	Stand end
12.7 ± 2.3%	8.4 ± 2.5%	7.8 ± 1.7%	7.3 ± 1.4%	16.4 ± 3.5%
***Probability***	**Stand end**	**Sit flat**	**Sit slope down**	**Slope back**
**Stand start**	0.133	**0.002**	**<0.001**	**0.002**
**Sit flat**	**0.001**	-	0.322	0.513
**Sit slope down**	**<0.001**	0.322	-	0.616
**Slope back**	**<0.001**			

Probabilities of <0.05 are in bold type.

**Table 9:** **Short notes un-tongued – external oblique (% maximum activity, mean ± s.e.m. n = 20)**

Stand start	Sit flat	Sit slope down	Slope back	Stand end
22.5 ± 4.5%	10.2 ± 2.3%	13.7 ± 4.4%	11.3 ± 3.5%	20.1 ± 4.2%
***Probability***	**Stand end**	**Sit flat**	**Sit slope down**	**Slope back**
**Stand start**	**0.026**	**<0.001**	**0.013**	**0.001**
**Sit flat**	**<0.001**	-	0.226	0.059
**Sit slope down**	**0.002**	0.226	-	**0.022**
**Slope back**	**0.008**			

Probabilities of <0.05 are in bold type.

**Table 10:** **Short notes un-tongued – rectus abdominis (% maximum activity, mean ± s.e.m. n = 20)**

Stand start	Sit flat	Sit slope down	Slope back	Stand end
16.8 ± 3.4%	10.0 ± 2.1%	10.3 ± 2.5%	9.9 ± 2.3%	23.2 ± 4.7%
***Probability***	**Stand end**	**Sit flat**	**Sit slope down**	**Slope back**
**Stand start**	0.092	**<0.001**	**0.001**	**0.004**
**Sit flat**	**<0.001**	-	0.367	0.467
**Sit slope down**	**<0.001**	0.367	-	0.311
**Slope back**	**<0.001**			

Probabilities of <0.05 are in bold type.

For both muscles, EMG activity for un-tongued notes was significantly greater than for tongued notes in all positions except one (Table 11). This was for the external oblique muscle when sitting on a flat seat (Sit flat). The difference between tongued and un-tongued notes was greatest when standing.

**Table 11:** **Probability of differences between muscle activity during sforzando tongued versus un-tongued notes (n = 20)**

	Stand start	Sit flat	Sit slope down	Slope back	Stand end
**External oblique**	**0.007**	0.092	**0.047**	**0.020**	**0.009**
**Rectus abdominis**	**0.006**	**0.044**	**0.043**	**0.011**	**0.001**

Probabilities of <0.05 are in bold type.

### Experiment 3-playing a study piece

**Figure 5:**
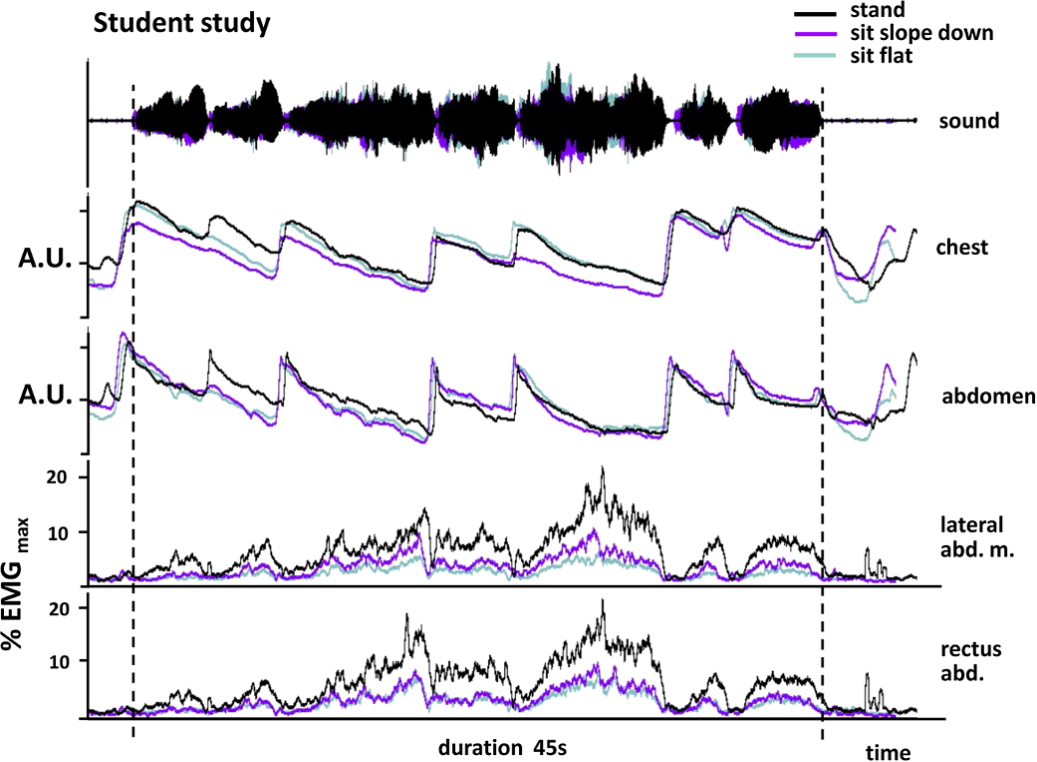
**A comparison of the level of abdominal muscle activity and movement of the chest and abdominal wall during performance of a trumpet study in different postures by a student player.** The electromyographic signals from both the external oblique and rectus abdominis are markedly lower when sitting on a flat seat or downward sloping seat than when standing. A.U. arbitrary units.

The overall activity of the muscles while playing the study piece was estimated as the area under the electromyographic traces after root mean square transformation. When players were sitting on a flat or a sloping seat, activity in the rectus abdominis and external oblique muscles showed a 32–40% reduction in activity when compared to standing (e.g. Figure 5) that was highly significant statistically (Table 12). There was however no statistical difference between the two sitting positions (p = 0.45 for the external oblique and p = 0.18 for rectus abdominis).

The study piece was chosen so that it could be repeated within a short space of time in the different positions and so did not make extreme demands on the players. It therefore did not match the intensity that is sometimes required in performance; the example in Figure 5 has a peak muscle activity of only about 20% of MVC.

**Table 12:** **Studies - muscle activity at Sit flat and Sit slope down measured as the area under the trace (RMS) expressed as a percentage of the level seen during standing (n = 8)**

	External obl. Sit slope down	External obl. Sit flat	Rectus abd. Sit slope down	Rectus abd. Sit flat
**Mean ± s.e.m.**	68 ± 5.7%	60 ± 6.3%	68 ± 4.6%	61 ± 5.0%
**Probability**	**<0.001**	**0.002**	**0.003**	**0.003**

Probabilities of <0.05 are in bold type.

#### Case study

**Figure 6:**
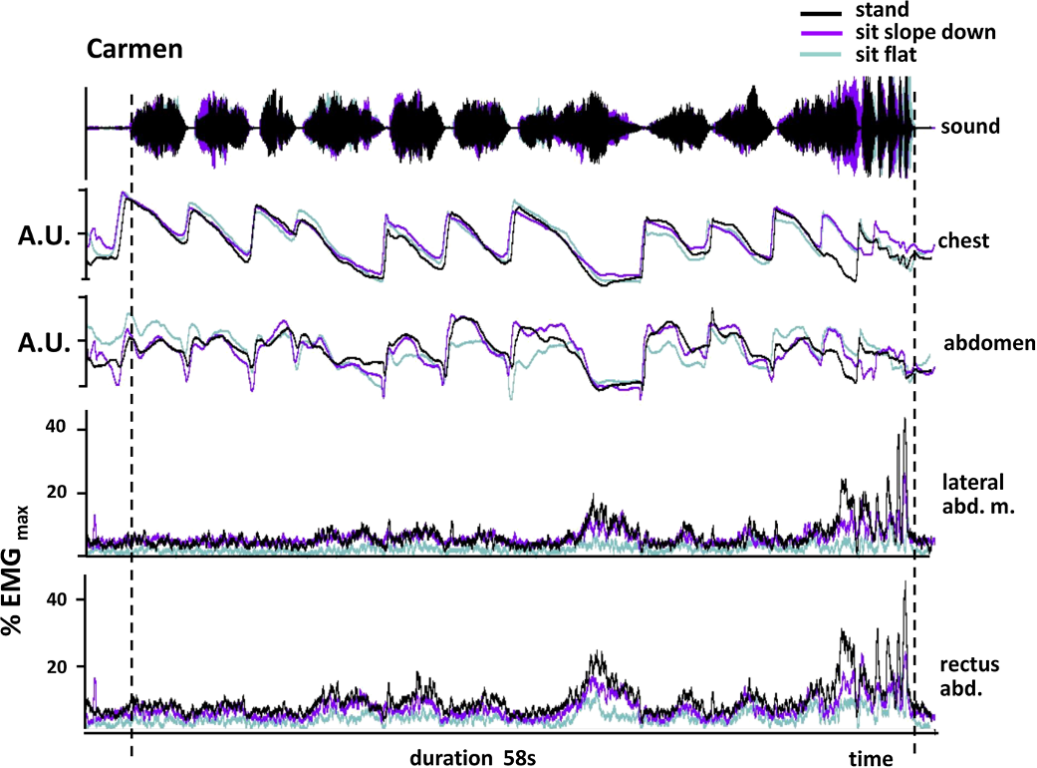
**A comparison of the level of abdominal muscle activity and movement of the chest and abdominal wall during the performance an orchestral excerpt (from the prelude to Act 1 of Carmen) in different postures by a professional player.** This is low in the trumpet range and overall, levels of muscle activity are quite modest. They are greatest when standing and lowest when sitting on a flat seat. Sitting on a sloping seat generates a level of muscle activity that is close to that of standing. The pattern of thoracic abdominal movement is similar in all positions and shows no evidence of consistent differences in degree.

**Figure 7:**
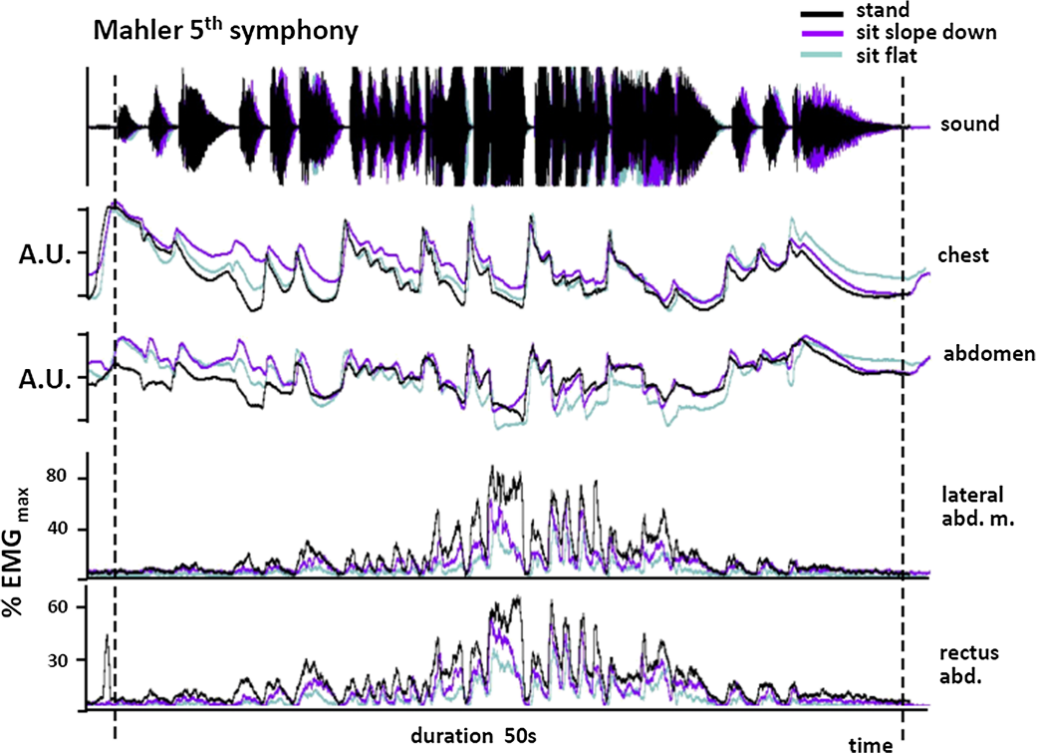
**A comparison of the level of abdominal muscle activity and the movement of the chest and abdominal wall during the opening bars of the first movement of Mahler’s 5th symphony performed in different postures by a professional player.** Muscle activity reaches much higher levels than for the excerpt in Figure 6 reflecting the higher pitches and sound intensity generated. Activity is greatest when standing and lowest when sitting on a flat seat. Sitting on a sloping seat results in an intermediate level of activity. There is no consistent difference between chest and abdominal movement in the three postures.

For illustrative purposes therefore, we made similar recordings of abdominal muscle activity during professional level performance by an elite trumpeter playing three orchestral excerpts. This provided an opportunity to make a qualitative comparison of the effect of playing at different intensities. The Carmen extract was the lowest in pitch and volume, and activity in the two muscles peaked at 30–35% of MVC. For the Mahler extract, peak activity was 70–80% MVC and for the Strauss, the peak was 100%. The results confirmed that, as in the pieces played by the students, overall activity in both the rectus abdominis and external oblique muscles was reduced when sitting on a flat or sloping seat. However in line with the results in experiment 2, sitting on a downward sloping seat in this more realistic playing scenario resulted in levels of abdominal muscle activity more clearly intermediate between sitting on a flat seat and standing for all three orchestral excerpts (Table 13) despite the fact that they demanded different absolute levels of muscle activity (Figures 6 and 7).

**Table 13:** **Case study (elite trumpeter) – muscle activity measured as the area under the trace (RMS) expressed as a percentage of the level seen during standing**

	Carmen (low range)		Mahler (middle range)		Strauss (upper range)	
	Rectus ab.	Ext. obl.	Rectus ab.	Ext. obl.	Rectus ab.	Ext. obl.
**Sit slope down**	80	94	67	81	84	80
**Sit flat**	51	82	45	66	56	32

## Discussion

### The effect of posture on respiratory function

Though most previous investigations into the effect of posture on lung function compare standing with lying in a prone or supine position, two studies present results that are relevant to ours. One compared lung function when standing with sitting erect on a flat seat in healthy non-obese subjects of both sexes (Lalloo et al. 1991). Like us, they found no statistically significant difference between the two postures for VC, FVC, FEV1 or PEF except that in females, FEV1 was reduced when sitting. Another study also found no statistical difference in VC between standing and sitting (Kera and Maruyama 2005), however Lalloo et al. (1991) noted a consistent but non-significant reduction in the VC, FVC and FEV1 in sitting. Quanjer et al. (1993) reported that in middle-aged (but not young) people, VC is 70 mls less when sitting compared to standing. They suggested that this emerges with age as a result of the reduction in physical activity and consequently of lung function. In our young adult subjects there also appeared to be a small non-significant reduction (26 mls) in VC when sitting on a flat seat but even if a further study involving more subjects rendered this statistically significant, the size of the reduction would make little impact on performance as it represented only 0.6% of mean VC when standing.

One factor that might influence spirometric values is arm position. Our subjects held the spirometer to their mouths with one hand while the other hung loose. Presumably other studies of respiratory function also did this, though details are not provided. A reduction in inspiratory capacity is seen as both arms are rotated progressively upwards, though it becomes statistically significant only when the they are raised above the horizontal (McKeough et al. 2003). In order to support instruments such as the trumpet and trombone, players must elevate their arms though they remain well below shoulder height. Brass players may however protract the shoulders which also appears to reduce VC and FVC (Ghanbari et al. 2008). This posture can develop as the pectoralis major and the anterior deltoid muscles strengthen and shorten over time as a result of supporting the instrument for prolonged periods (Price and Watson 2011; Watson 2009). Players may even consciously adopt this posture in the mistaken belief that it increases VC. Classical singers (in whom it is sometimes known as the “gorilla posture”) sometimes also use it in the belief that it improves breath control (Miller 1997) but there is no evidence to support this.

Publications dealing with the ergonomics of musical performance frequently discuss the use of chairs with sloping seats (Paull and Harrison 1997; Horvath 2010; Rosset i Llobet and Odam 2007). Diverse opinions on optimal sitting posture have been expressed in various orthopaedics texts (see Claus et al. 2009) though generally without reference to objective evidence, however it has been reported (Harrison et al. 1999; Keegan 1953) that an angle of 135° between the trunk and the thighs reduces the pull of the thigh muscles on the pelvis and allows the lumbar lordosis to assume what was defined as the neutral position (similar to that of standing). This angle would be impractical for musicians on a smooth seat as the buttocks would tend to slide downwards. Several types of ergonomic chair with downward sloping seats are commercially available (e.g. kneeling or saddle chairs). The seats of kneeling chairs may slope by as much as 20° (Bettany-Saltikov et al. 2008) creating an angle between the trunk and thighs of 110°, while in saddle chairs in which a raised pommel-like central ridge prevents forward slippage of the body, the angle between trunk and thigh may exceed 125° (Gandavadi and Ramsay 2005). Kneeling chairs do restore a moderate degree of lordosis in comparison to the kyphosis that is a typical consequence of sitting on flat seats, but this remains well below that of erect standing (Bettany-Saltikov et al. 2008). Musicians sometimes adapt the chairs provided with by placing blocks under the back legs or using a wedge shaped cushion (Paull and Harrison 1997). The cushions typically have a slope of 11–22 degrees (representing a trunk to thigh angle of 101–112 degrees) but this likely to be reduced by compression when the player sits on them. Alternatively, the player may sit on the front edge of a flat seat, holding the trunk erect and allowing the thighs to slope downward, though the degree to which this is possible will depend on the relationship between seat height and leg length. A number of chairs designed specifically for musicians are also available. In most, the slope of the seat is quite modest though one has a maximum downward slope of about 15° (allowing a trunk to thigh angle of 105°). In chairs with a gentler slope, rounding the front edge of the seat allows the thighs to slope more steeply when the player sits forward. When sitting erect on sloping seats, greater activity is required in the postural muscles of the back and so it make take time to acclimatise and strengthen them, particularly if there is no lumbar support (Claus et al. 2009; Gandavadi and Ramsay 2005). The degree of lordosis will depend on how erect the trunk is held by voluntary muscle activity. In orchestras, bands or other ensembles, performers often play sitting for several hours a day but unless they are physically active, core stability (dependent on distal limb and trunk musculature) may be relatively weak (Ackermann et al. 2002; Kava et al. 2010).

Whatever the postural advantages of a downward sloping seat, sitting with a trunk to thigh angle of 115° was the only posture in our study that produced a significant difference in the maximum duration of held notes compared to standing; however this was not an enhancement, but a reduction of about 8% which might be correlated with the near significant reduction in VC compared to standing. There was no significant reduction in FVC, but playing a note of maximum duration puts a greater premium on lung volume than airway pressure. It is possible that one factor in this may have been that players felt unstable in this unfamiliar posture. Muscle activity in the external oblique, though not rectus abdominis, was significantly greater on the downward sloping seat than on a flat seat, though this did not translate into a consistent difference in the change of abdominal circumference during the exhalation. Neither was there a difference in the level of muscle activity supporting short blasts of sound. However, as the maximal muscle activity measured during the long notes came right at the end when the last of the air was being expelled from the lungs, the demands made by these two tasks were very different.

### Sitting posture and abdominal muscle activity

One review of sitting posture suggests that intervertebral disk pressure and postural muscle activity is minimised in a chair with lumbar support, whose back slopes at 120° and whose seat slopes upward by up to 10 degrees (Harrison et al. 1999). It is clear from our spirometric data that reclining significantly reduces both vital capacity and forced vital capacity. The brass musicians found trying to play in this posture very uncomfortable so to avoid any risk of injury, we reduced the slope of the chair back for experiment 2. Standing in a stooped position or sitting on a backless seat in a forward slumped posture also reduces activity in the lateral abdominal muscles compared to standing (O’Sullivan et al. 2002; Snijders et al. 1995) and our experiences with upward sloping seats (which also bring the angle between thigh and trunk to below 90°) suggest that this will reduce both vital capacity and forced vital capacity.

An important factor in respiratory biomechanics is that when standing, abdominal wall tension is 30% greater than when sitting (van Ramshorst et al. 2011) and intra-abdominal pressure is increased by 20% (Cobb et al. 2005). Abdominal wall tension is greater during inspiration than expiration and rises even higher during the Valsalva manoeuvre (van Ramshorst et al. 2011). When playing standing up, the need to overcome abdominal wall tension in order to drive the diaphragm upwards would explain why abdominal muscle activity is greater (even though the reduction in abdominal circumference was smaller) than when sitting. Though brass players are often exhorted to sit as if standing, i.e. with an erect back (Frederiksen 1996) this will not in itself result in an abdominal wall tension as great as when standing. The reason why abdominal wall tension is greater in standing is not entirely clear, however the angle of the pelvis causes the lumbar spine to curve into lordosis which will both push the abdominal contents forward and stretch the abdominal wall through a compensatory backward tilting of the thorax to keep the centre of gravity correctly aligned over the feet. On a flat seat, the pelvis rotates backwards on the ischial tuberosities, flattening the lumbar spine. The thorax falls forward, reducing the tension in the anterior abdominal wall. Players who sit at the front of the seat with the thighs sloping downwards, do so in order to rotate the pelvis forward towards the standing position and so increase the lumbar lordosis. However it may be insufficient to restore abdominal wall tension even if the spine is consciously extended and the thorax tilted back. Whether sitting in this way would provide any advantage for respiration is unclear. Our spirometric data indicate that only sitting in a chair with a sloping back or an upward sloping seat had a consistent effect on the parameters measured but this was a negative one. Even so, the differences only amounted to a few percent and neither produced a reduction in maximum note duration.

It has been noted that abdominal wall tension is significantly higher in men (31%, P < 0.0001) than in women (van Ramshorst et al. 2011). This has been attributed to some unknown aspect of physique, however our results for muscle activity during playing tasks showed no obvious gender effect though the relatively small sample size and confounding factors such as height and weight, mean that it was not open to statistical evaluation.

Another factor that may be important is body type. All of our subjects were young with a mean body mass index of around 25 (range 18–40). Van Ramshorst et al. (2011) failed to find any correlation between abdominal wall tension and body mass index (BMI), though they noted that the small BMI range among their subjects (17–28, mean 22.7) might have limited the ability to detect any relationship. Cobb et al. (2005) did find a significant correlation between BMI and intra-abdominal pressure for standing, either with or without the Valsalva manoeuvre, though not for sitting. The raised airway pressure required for brass playing can have a similar effect on abdominal pressure to the Valsalva manoeuvre even if the larynx is not locked. Closure of the glottis is a relatively common fault in brass players and has a variety of negative consequences both elevating airway pressure unnecessarily (Stasney et al. 2003) and making articulation uncertain (Watson 2009) and is generally discouraged with the instruction to “play with an open throat”.

### Abdominal muscle activity during breathing

Some caution is needed in interpreting the significance of different levels of EMG activity (Sundberg et al. 1991). The key question is whether an integrated signal such as the root mean square derivative used here, is linearly related to the force generated by the muscle. Though this is a quite a complex issue, it has been suggested that the relationship is close to linear for isometric contractions (Roberts and Gabaldon 2008), and as the length of the abdominal wall muscles changes only slowly during brass performance, this condition would largely be satisfied in the current experiments. However it is clear from Figure 3, that though there is a steady decline in abdominal circumference right from the start of a long exhalation, the abdominal muscle activity responsible is not detectable by surface electrodes until sometime into the breath. This may be one reason why the relationship between the EMG amplitude and muscular force appears to be strongly non-linear for low levels of activity (Roberts and Gabaldon 2008). While it would be advantageous to use implanted wire electrodes for this type of study, few of our subjects would volunteer for this. Another factor that needs to be considered is that the electrodes record activity only from muscle fibres that are relatively nearby. The external oblique is a wide sheet of muscle and rectus abdominis is composed of a number of muscular segments linked in series by tendinous junctions, so a single pair of electrodes will sample activity in only a limited region. During speech and singing much higher levels of activity have been reported in the inferior regions of lateral abdominal muscles than in the superior regions (Hoit et al. 1988; Watson et al. 1989). Our recording sites were relatively low but as the airway pressures required for brass performance are much higher than for singing, it cannot be assumed that the pattern of activity in the abdominal muscles will necessarily be similar.

Surface electrodes over the external oblique muscle will record relatively little activity from the internal oblique and transversus abdominis. Using implanted electrodes, (de Troyer et al. 1990) found that the transverse abdominis was engaged simultaneously with the external oblique during spoken counting and isovolume manoeuvres. Using implanted electrodes, Kera and Maruyama (2005) recorded from both the internal and external oblique muscles during a variety of breathing tasks and showed that their activity tended to fluctuate in parallel. Together, these two sets of observations suggest that the three sheets of lateral abdominal muscles often act in concert during respiration (de Troyer et al. 2005). Of particular relevance to our study, is the observation by de Troyer et al. (1990) that in many subjects, activity began almost simultaneously in tranversus abdominis, external oblique and rectus abdominis during several types of breathing including exhaling against a closed glottis. The latter would be similar to brass players exhaling against the resistance of the embouchure. Nevertheless, there remains some controversy over the pattern of abdominal muscle activity during forced exhalations. Though the lateral abdominal muscles are consistently reported to be strongly activated in speech-related breathing, bearing down on a held breath (straining), singing or coughing, all of which might be expected to share some features with wind playing, several studies found little activity in rectus abdominis (Basmajian 1978; Floyd and Silver 1950; Hoit et al. 1988). By contrast, others reported that such divergent activities as flute playing, which requires relatively low breath pressure (Pawlowski and Zoltowski 1987; Watson 2009) and trumpet playing, which needs much higher pressures, both involve a major recruitment of rectus abdominis (Berger 1968; Cossette et al. 2008; de Sousa and Furlani 1974; de Troyer et al. 1990, 2005). This was also our experience. It should be noted however that the very different patterns seen for counting in two of these studies suggests that the protocols were not equivalent (de Troyer et al. 1990; Hoit et al. 1988). The notion that the external oblique muscle is more important than rectus abdominis during forceful expiration has been perpetuated in the literature on brass players, though the study on which it is based involved only a single trumpeter and presents no actual data (Bejjani and Halpern 1989; Draper et al. 1960).

The level of muscle activity required for the relatively moderate playing tasks (sforzando blasts and the technical study) played mezzo forte by our student subjects typically required abdominal muscle activity of around 20–60% of MVC. Occasionally the students coughed spontaneously during recordings and this was supported by brief but much higher levels of activity (up to 100% MVC). The playing tasks were therefore not taxing in comparison to the level of activity that could easily be generated in normal behaviours. Playing orchestral excepts at performance volume in our case study required peak bursts of muscle activity at 80–100% of MVC for much longer durations than for a cough though the limiting factor will generally not be abdominal muscle fitness or strength, but that of the embouchure. For the trumpet, the increase in the volume of the sound produced by a doubling of airway pressures falls rapidly with pitch from around 15 dB in the low to mid register to only 3 dB in the highest (Fletcher and Tarnopolsky 1999). This explains why loud orchestral playing in the upper register requires such high abdominal muscle activity with small increments in volume requiring progressively greater increases in pressure.

### Study limitations

A common shortcoming of the majority of studies of the physiology of musicians so far published is the limited availability suitable numbers of subjects. For this reason, most involve conservatoire students. For greatest consistency these should play the same instrument and be at a similar level of development but this restricts the number of suitable subjects may volunteer. For simple standardised playing tasks (long notes, sforzando blasts) we therefore pooled data from students playing different instruments, but for more realistic tasks (i.e. real music) it was necessary to concentrate on the single instrument that offered the most subjects. Though we were able to show statistical differences between standing and sitting, we could not do so for the two sitting positions even though the mean values for abdominal muscle activity were consistently different. A number of the probabilities we calculated were close to significance and might have become so with a larger sample. A second issue is that the respiratory demands made of professional players are very considerable and our students had limited experience of this type of playing. Our case study with the professional player suggested that when respiratory demands are high in terms of airway pressure (equating to higher pitch and sound intensity), the differences in the level of abdominal muscle activity between sitting on a flat or downward sloping seat may be much larger than in our student study. This warrants further investigation but would depend on the recruitment of an appropriate number of elite players.

### Conclusion-vocational significance

Though spirometry requires a different type of breathing than brass playing (i.e. there is no resistance to exhalation) it nonetheless removes a number of confounding factors such as differences in the in the way particular instruments are supported, and isolates the potential influence of posture alone. One widely used pedagogic text on breathing in brass players suggests that sitting “seriously reduces the expansion area of the thorax” (Steenstrup 2004). While we did not observe any difference in the degree of thoracic expansion, the spirit of this idea may be supported by the significant or near significant reduction in vital capacity, forced vital capacity and maximum note duration when sitting on a flat or sloping seat compared to standing. However this amounted to a few percent at most and so is unlikely to have a major impact on performance. The widely held notion (Frederiksen 1996; Steenstrup 2004) that it is possible by sitting erect to create a lumbar posture that is comparable to standing (as sitting on a downward sloping seat was intended to do in our study) and that this is reflected in breathing patterns, was not upheld for spirometry or for moderate playing tasks carried out by the students, but when playing at high intensity, using a sloping seat may create a situation that is truly intermediate between sitting on a flat seat and standing.

While some of the differences in muscle activity between standing and sitting are no doubt due to constraints directly imposed by posture, another important factor may be the change in subjective sensations perceived by the player. Greater abdominal wall tension when standing gives the impression that more work is required during exhalation (though less for inhalation) and we have seen that abdominal muscle activity is indeed greater. However the inward movement of the abdominal wall is smaller when standing, which may appear counter-intuitive to players and teachers. The reduced abdominal muscle activity when sitting may lead to greater embouchure muscle activity in a bid to compensate for the subjective impression of reduced air support. As we did not measure oral pressure, we cannot say whether this was in fact reduced and this would be worthy of further investigation. As players perform both standing and sitting, but generally stand when practicing, our results suggest that it would be advantageous to vary their practice routines to incorporate both positions. They might also benefit from experimenting with sloping seats but they would need to adjust to the altered sensations this generates and be prepared to develop their core stability (postural muscle strength).

We observed that though tongued notes required less muscle activity than untongued ones, their onset was less tightly controlled. Developing control of support through breath attack practice as has been advocated by a number of writers on brass technique and may be advantageous for developing control of breath support (Fox 1974; Tuckwell 1978).

Preconceptions and stereotypes typify brass playing and teaching which has traditionally been dominated by males, but professional orchestras of international calibre are now appointing female brass players to principal positions and they make up an increasingly large proportion of brass students in conservatoires. Several female brass players also have prominent international solo careers. Their gender does not therefore present any insuperable barrier to elite performance despite physiological differences with men, some of which are well known (e.g. average height and hence vital capacity, muscle development) and others less so- (abdominal wall tension - van Ramshorst et al., 2011). Given the constraints of sample size and various confounding factors in our studies, we were not able to investigate this specifically, though we saw no obvious differences attributable solely to gender. This is a subject that would benefit from further investigation.

Cross fertilization of ideas is common in the pedagogic literature of different musical disciplines and because there is so much more written about breathing in singers, brass players may turn to this literature as a source of information. Airway pressures in brass performance are much higher than in singing and so it cannot be assumed that the pattern of abdominal muscle activity of chest and abdominal movements will be similar. That is why it is vital to seek objectively validated information relevant for each group of performers. However there are common trends emerging across disciplines. One is that individual variation in respiratory strategies is much greater than some teaching doctrines acknowledge. This is frequently hidden because what performers actually do may not match what they think they are doing. Classical singers (and indeed wind players) may be taught to keep the chest expanded when performing, but its circumference always declines considerable during exhalation regardless of what performers believe (see Watson et al. 2012). Amongst our brass players, we also saw considerable variation, for example in how soon after the onset of a long note, inward abdominal wall movement was initiated. This might indicate that different strategies suit particular individuals, perhaps because of variations in body morphology. Whatever the reason, it emphasises the importance of encouraging musicians to take a scientific approach to investigating vocationally important aspects of physiology rather than perpetuating ideas that though widespread, have never been objectively validated. It also suggests that teaching should not be overly dogmatic, but include an awareness of anatomical and physiological differences between individuals and that the same ends may be achieved by a variety of means.

## Methods

Three separate experiments were carried out.

1. Spirometric measurements taken when standing were compared to four sitting positions (Figure 1). These were; a) sitting in an upright chair with a horizontal seat, feet on the floor and with the trunk at 90° to the thighs [here referred to as “sit flat”], b) sitting with the trunk vertical on a seat that sloped downwards so that the angle between the trunk and thighs was 115° [“slope down”], c) sitting on a horizontal seat with the back reclining so that the angle between the thighs and the trunk was 122° [“slope back”] and d) sitting with trunk vertical and the thighs sloping upwards to make an angle of 65° with the trunk [“slope up”]. Ergonomic chairs with kneeling or saddle designs require a trunk to thigh angle of 110°–125° (Bettany-Saltikov et al. 2008; Gandavadi and Ramsay 2005). These have a pommel or knee support to prevent the sitter slipping off. We chose 115° for “sit slope” as the steepest angle that could comfortably be maintained without slipping. Positions c) and d) were extreme positions in which the biomechanics of breathing should be considerably altered. The liver (weight around 1.5 kg in the adult male) is attached to the underside of the diaphragm and gravity contributes to its descent during inhalation. In more supine positions such as “slope back”, this effect is reduced (Hoit 1995). The angle used is also said to minimise intervertebral disk pressure and postural muscle activity (Harrison et al. 1999). Reducing the angle between the trunk and thighs to 65° (“slope up”) would be expected to restrict anterior abdominal wall expansion and restrict the descent of the diaphragm. The subjects were 30 undergraduate students (13 males, 17 females of Caucasian origin) from the Royal Welsh College of Music (RWCMD) and Drama and Cardiff University, age 20.7 ± 1.0 yrs (mean ± s.d.), mean BMI 23.3 ± 3.6. Ten were music (brass) students and 20 were science and humanities students but as there was no significant difference between the spirometric data from two groups, this was pooled.
2. In the second experiment, abdominal respiratory muscle activity was monitored using electromyography [EMG]) and the changing circumference of the chest and abdominal walls was recorded by inductive plethysmography. Subjects were first asked to play two notes at the same constant pitch and volume (mezzo forte) for as long as possible, taking as much time as they wished to recover in between. To make the notes equivalent for each instrument, the third harmonic was used (open fingering for valved instruments, first position for trombone). The second task demanded more abrupt and forceful muscle activity to generate a series of 8 short sforzando notes at the same pitch (forced blasts) each of 1 s duration, separated by three seconds of silence. A breath was taken prior to each note and the exercise was paced with a metronome. Alternate pairs of notes were played tongued and un-tongued. Both tests were carried out while standing and then repeated in three sitting positions. Two were positions a) and b) from experiment 1 (“sit flat” and “slope down”) but for third (“slope back”) the angle between the thighs and the trunk was reduced to 115° as the players complained of discomfort when reclining at the angle used for spirometry. The upward sloping seat was not used as it was not a realistic playing position. Finally, the tasks were carried out once more in the standing position, as a control for the possibility that increasing fatigue might be a progressive factor during the test series. The subjects were 20 brass students from RWCMD, 11 males and 9 females; mean age 20.0 ± 0.9 yrs, BMI 25.3 ± 5.4. Six played the trumpet, 5 the French horn, 3 the euphonium, 2 the tenor horn, 2 the cornet, and 2 the trombone.
3. In the third experiment, EMG and inductive plethysmography recordings were made during a more realistic playing task. This was a study for trumpet lasting about 50 seconds (technical study no. 6 “Breath Control” from a set written for first year brass students at RWCMD by C. Mowat). It was performed mezzo forte with the tempo regulated by metronome. It was played standing and in the “sit flat” and “slope down” positions already described then repeated standing. The subjects were trumpet players only. This was to avoid the issue of whether studies played on different instruments were equivalent in their respiratory demands, however it meant that the sample size was reduced to eight 8 (5 females and 3 males; mean age 19.6 ± 2.2 yrs, BMI 22.1 ± 2.7 (mean ± s.d.)).

In addition, as an illustrative case study, an experienced professional male trumpeter (section leader of a national orchestra) performed three orchestral excerpts in different pitch ranges. These were a) the prelude to act 1 of Carmen by Bizet (low pitch range; duration 75 s), b) the beginning of the 1st movement of Mahler’s 5th symphony (middle range; duration 50 s), and c) “Auf dem Gletscher” from Richard Strauss’s Alpine symphony (high range; duration 12 s). All were played with appropriate dynamics and at a volume suitable for stage performance and in the same three postures used by the student trumpeters.

### Ethics

All experiments were carried out with informed consent and according to the Helsinki declaration, and were approved under the local ethical procedures of the School of Biosciences, Cardiff University.

### Spirometry

Spirometric performance was tested using a MicroMedical Spiro-USB Spirometer (Cardinal Health Ltd, Kent, UK) and analysed with Spida5 software (Cardinal Health, Kent UK). The parameters examined were Vital Capacity (VC), Forced Vital Capacity (FVC), Forced Expiratory Volume in the first second (FEV1) and Peak Expiratory Flow (PEF). Three replicates were taken and the best performance was used for the analysis. To control for the effects of height and gender, the values for the parameters measures were expressed as percentages of predicted values. These can vary depending on ethnic origin so were used ECSC algorithms, which are relevant for Caucasians (Quanjer et al. 1993) and commonly employed for clinical evaluations. All subjects were classed as clinically normal by the software used, none was suffering from any respiratory complaint and while some had previously been diagnosed with asthma, none used an inhaler routinely or on the days of data collection.

### Inductive plethysmography

**Figure 8:**
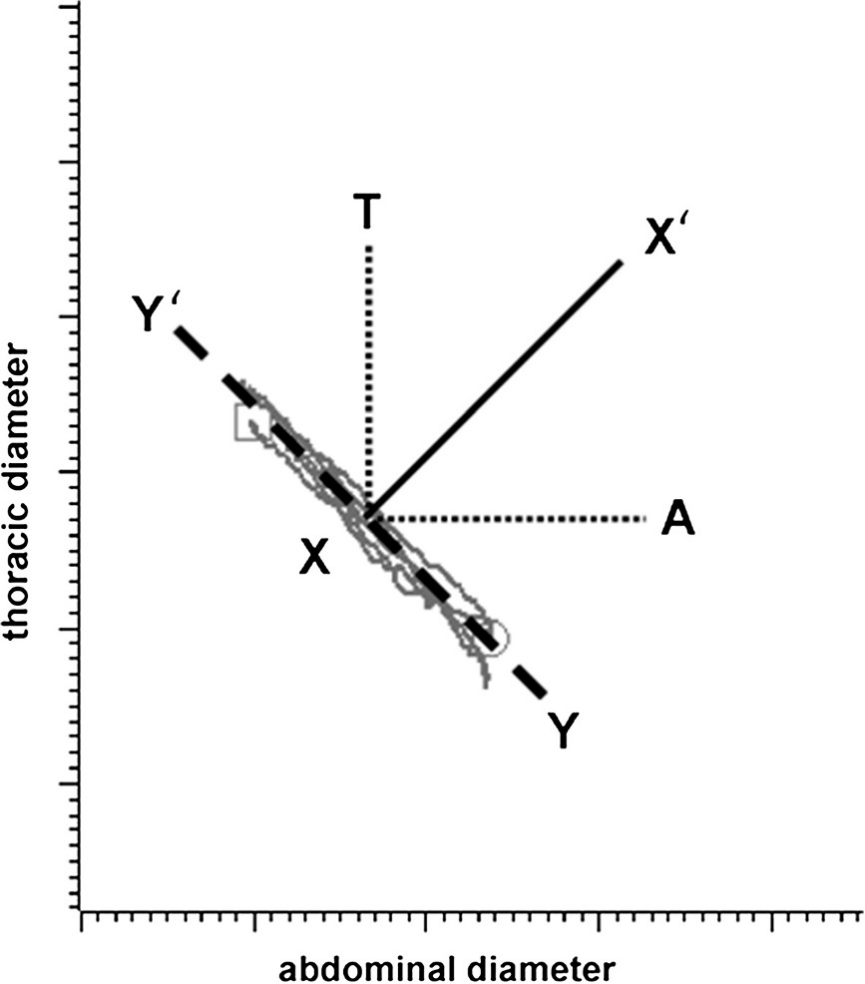
**Each subject was asked to carry out an isovolume manoeuvre and as shown here in a Konno Mead diagram, whose axes were adjusted to give a slope of -1 for the trace.** The same scales were used for the x and y axes of the experimental traces for that subject. Further explanation of the figure is provided in the Methods.

Inductive plethysmography was carried out using an Inductotrace system (model 10.9000, Ambulatory Monitoring, Arosley, NY, USA). Two inductive respibands were used, one placed around the chest at the level of the axilla and the other around the abdomen, below the lowest rib but above the iliac crest. The analogue signal, which is a function of circumference, was digitised using a CED 1401 A/D converter and displayed with Spike6 software. Changes in chest circumference were also plotted against those of abdominal circumference as Konno-Mead diagrams (Konno and Mead 1967). Plethysmograph recordings were made of isovolume manoeuvres and of breathing during the activities already described. Analyses of the data was based on that of Hixon et al. (1973). For each subject, the x and y axes of Konno-Mead diagrams of the isovolume manoeuvres were manipulated to produce a slope for the traces of -1 (indicated by the dashed line Y-Y’ in Figure 8). This represents chest and abdominal movements that together result in no volume change. The same scaling was applied to the axes of all other Konno-Mead plots from the same subject. The slopes of the exhalation phases were measured relative to a line X-X’ (slope +1) that lies at 90 degrees to Y-Y’. This represents changes in volume that are brought about by equal changes in circumference of the thorax and abdomen. Slopes greater than that of the line were designated as positive and those less, as negative. The line T-X (+45°) represents volume changes that are entirely due to reductions in thoracic circumference, and A-X (-45°) volume changes due solely to reductions in abdominal circumference.

### Electromyography (EMG)

Muscle activity was recorded from the external oblique and rectus abdominis muscles using a CED 1902 amplifier (Cambridge Electronic Design, Cambridge, UK) connected to a CED 1401 A/D converter. After skin preparation with alcohol wipes, pairs of self-adhesive bipolar silver/silver chloride surface electrodes (Kendall Medi-Trace 100, Tyco Healthcare Group, Mansfield, USA) were attached to the skin approximately 2 cm apart, parallel to the predicted direction of the muscle fibres. The electrodes for rectus abdominis were placed about 2 cm from the midline with the upper one just below the level of the umbilicus. Those for the external oblique were placed at a similar level 2 cm medial to the anterior superior iliac spine. The latter pair of electrodes are likely to experience some cross talk from the internal oblique or transversus abdominis muscles as they will almost certainly also be active during the playing tasks (Agostoni and Campbell 1970; Hoit et al. 1988). The signal therefore represents the activity of the lateral abdominal muscles as a whole, but dominated by the external oblique. The ground electrode was placed over the spinous process of the seventh cervical vertebra. In addition to monitoring electromyograph activity during playing, the activity during maximum obtainable voluntary contraction (MVC) of the each muscle was also recorded. The y-axis scale of the EMG traces in the figures are expressed as a percentage of this value. The EMG signals were sampled at 1 kHz and displayed on a computer using Spike6 software (Cambridge Electronic Design, Cambridge, UK) which allowed further conditioning and analysis. A high band pass filter (90 Hz) and a 50 Hz notch filter were applied in order to eliminate movement artefacts and electrical interference, and reduce cardiac signals where present. For quantification and further analysis, the raw EMG trace was processed using a root mean square (RMS) algorithm with a time constant of 100 ms. For sustained notes (experiment 2), activity in the two muscles rose progressively with time and the maximum amplitude attained was measured. This occurred at the end of the note. For short notes (whose duration and frequency were controlled using a metronome) the maximum of the RMS trace was measured and averaged over the number of trials (two for the sustained notes and four for the short notes). In experiment 3, which involved playing pieces of music, the area under the RMS trace was used as an indicator of overall muscle activity. A metronome was used to ensure that the duration of the piece remained constant and allow trace overlay for comparison. Sound was recorded with a Shure C606 microphone placed 1 m from the player. The signal was passed through an SP-24B preamplifier (Maplin, Rotherham UK) and then to the CED 1401. The voltage output of the sound trace was monitored to ensure that on repetition, the pieces were played at the same volume.

### Statistics

Statistical analysis was carried out using Minitab v. 16 (Minitab Inc., State College, Pennsylvania, USA). An Anderson Darling Normality test was performed on each data set, and if it was normally distributed a 1-tailed paired *t*-test was applied to compare sitting postures with standing. If the data failed the Anderson Darling test, Wilcoxon’s Signed Rank test was used.

## Abbreviations

FEV1: Forced expiratory volume in the first second
FVC: Forced vital capacity
MVC: Maximum voluntary contraction
PEF: Peak expiratory flow
VC: Vital capacity.
